# Ice-Houses

**Published:** 1866-04

**Authors:** 


### ICE-HOUSES.

THE *American Agriculturist* says: "First, the ground selected must be dry, and out of the way of floods, if near a stream; for if water stands in contact with the ice, it will melt away almost 'like the morning cloud.' It is well to have the ice-house on the north side of a hill, or of a house or big tree. If close to the house, and a cool-room can be made between it and the house, that will be found very convenient, and the ice-house wall next the cool-room need not be made so thick as on the other sides; in fact, a double boarding, with an inch of space between, is enough. It is well to dig out the ground so as to set the house a little lower than the general level, and it may be several feet lower if convenient. The bottom ought to slope to the middle or to one side, and to be grouted; that is, laid with broken stones which are covered with hydraulic cement mortar, poured over and in among them, and smoothed off even on the surface. The inclination of the bottom should lead to a sealed drain, so protected that it can not be stopped up by accident, or by sawdust. It is important that the drainage of an ice-house, whether the bottom be cemented as we have described or not, should be perfect, and that a circulation of air should not take place through the drain. This is easily affected by having the end of the drain (a round tile) rise two or three inches in a cemented depression, or basin, and turning over it a common flower-pot with the hole stopped.

"A house 10 × 10, or 12 × 12 feet, and eight feet from the bottom to the eaves, with a half-pitch roof, is about what is wanted on an ordinary farm, and will hold and keep more ice than is usually needed. The sides should be ten inches thick, the frame being of eight-inch uprights, of two-inch plank, set four on a side, (the end ones being a foot from the outside corners,) upon sills of the same width. The inside boarding should be of cheap inch stuff. The outside may be clapboarded, or boarded up and down and battened. Dry sawdust, planing-mill shavings, or dry spent tan-bark, may be used to fill in between the outer and inner boarding, and the filling should be settled down solid. The plates may be of two-inch plank; the rafters four on each side, of two-inch plank, six inches wide. They should be boarded outside and inside, and the space filled with shavings. The roof should be thatched or shingled, and the gable ends double boarded and filled like the sides. The door should be in one of the ends, four to six feet from the ground, and four feet high; and close to the peak there should be a sliding shutter for a ventilator. There should be a flooring not nailed down but laid firmly, to support the ice.

"The sides may rest on the grouting, or on a stone underpinning. When they are laid, they should have a coat of coal-tar all over, and when the house is done, sawdust stirred up with coal-tar should be filled into all the crevices and holes near the ground outside and inside, and earth heaped up around the sides and trodden down. Paint the sides with tar as high as the earth comes. How to fill an ice-house will be a subject for our December number.

"Straw Ice-Houses.—Where there is a great abundance of straw, ice may be preserved throughout the year, if packed in a compact mass and well covered with straw, perfect drainage being secured."

WANTED TO GIVE To whomsoever will procure sixty paying subscribers to Hall's Journal of Health for 1866, a Wheeler and Wilson sewing Machine, costing cash $56. This Machine will sew all kinds of fabrics, and is the cheapest and best manufactured of its kind. Specimen numbers will be sent post-paid for Ten cts. or

TO STUDENTS, The best and most complete edition of Webster's Dictionary, retailed at Twelve dollars, will be given to any one who will procure Twelve Subscribers, which many a person could do in an hour's time, who could not clear that much money in a month, in any way available to them. Or we will furnish a copy of all our publications, nineteen in number, for thirty subscribers.

GODEY'S LADY'S BOOK, Edited by Mrs. Sarah J. Hale and L. A. Godey, published monthly at the north-east corner of Sixth and Chestnut St. Philadelphia, Pa. for Three Dollars a year, is said by an exchange to be "The oldest, best and cheapest, for the best is always cheapest." The March No. contains Twenty embellishments, and about sixty different articles of reading matter. Back Nos. can always be supplied. Twelve copies are sent for one year for Twenty-eight dollars.

A VALUABLE BOOK. The Harper Brothers have just published an 8vo. of 300 pages, in handsome style, for $3.50, being A Text Book on Anatomy, Physiology and Hygiene, by Prof. John C. Draper of the University of New-York; for the use of Schools and families; with 170 illustrations; it is at once scientific, practical and popular, and well deserves to be made a text book in every public school, seminary, college and university; if this were done, the knowledge which would be imparted to the young and others, in reference to the laws of the human body, would not only prevent an incalculable amount of sickness, but would add several years to the average of human life. We will send the book post-paid to any subscriber who will forward four subscriptions to the Journal for 1866.

*The American Tract Society* of 28 Cornhill, Boston, and 13 Bible House, New-York, has sent us one of the sweetest little books it has ever published, "Polished Diamonds," by Rev. John Todd, D.D. Whoever has lost a darling child, whoever may have one to loose, will find in these few pages a comfort inexpressible. Its first chapter is headed "Mary Brace Todd," then "Grinding the Diamond," "The Pearl Oyster," "Crutches in the Garret," "The Funeral," "Heaven." Its first words are, "Just as the night begins to pass away, and the light of a new day to spread over the earth, the father stands at the fresh grave of his child with a little wreathe which he has woven to lay on that grave," and ends thus, "Dear reader! have I been able to throw one ray of light beyond the grave, or one beam of hope into your heart. I thank God if I have." This book is a well of water springing up unto everlasting life; and to every sufferer, to every one in trouble, it is a mine from which joy and gladness may be quarried, in every human sorrow, when there is hope in God. We will send a copy post-paid to every one who will send us three dollars for Two subscribers to Hall's Journal of Health for 1866—the offer will extend to the close of the year. "Precious Truths in Plain Words," contains sixty Tracts of two pages each, particularly adapted to family reading. Some of the subjects are, Hearers and Doers; Why we love God; Conflict here; Rest hereafter; True and False Peace; Always Rejoicing; Not Servants, but Sons; Wake or Die; The family in Heaven and Earth; Now or Never, &c. These two books will be sent for Three new subscribers.

"ENOCH RODEN'S TRAINING," 233 pages. This a reprint from the London Religious Tract Society, and every father who has a son whom he desires to restrain from evil ways, and to follow those paths which lead to usefulness and honor, and every youth who is about deciding what calling in life he shall pursue and who has an ambition to succeed, not merely in making money, but in becoming a respected and wealthy citizen, will derive invaluable practical lessons and hints from this very excellent book; as a means of introducing such books into families, the publisher will send the three, post-paid, for four new subscribers to Hall's Journal of Health for 1866; the offer to last during the year. This Society is doing a great and good work, and we heartily wish it commensurate success.

THE WORD OF PROMISE, 299 pages, being a hand book to the promises of Scripture, by Horatius Boner, D. D., shows that there is a promise making and a promise fulfilling God. How sweet to take the Bible and open it with such a thought filling the whole soul, making it the more ready to appropriate all its words of love and drink them in as the hunted hart drinketh in the cooling water brook! How much they loose daily, who fail daily to make the Holy Scriptures their ever dear delight. If any reader has not this delight in the Law of the Lord, it would be well for him to read just such a book, and others like it, especially as we know not what the coming summer may bring forth to any one of us; certain it is, that it will be the last on earth to some, and if they be not "saved," then have they lost a beatific immortality. To all we say, read such books as will not only inspire an unwavering confidence in the Bible, but will cherish a love and a habit of dwelling upon its truths, leaning upon its promise, and this will Dr. Bonar's delightful work help those to do, who want to do it.

FABLES FOR THE YOUNG FOLKS, by Mrs. Rosser, Original, may be safely and profitably placed on every family centre table. These fables will be a perfect delight to youthful readers, and cannot fail to make life-long impressions for good. The five books will be sent post-paid for five new subscribers to Hall's Journal of Health for 1866.. Five unusually excellent books without money; for a labor that almost any person might perform in half an hour, thus doing good all round.

CATARRH. The three advertisements in March, of Catarrh will save us the trouble of answering letters, which come to us from every quarter, inquiring as to the respective merits of the persons who advertise to treat the disease; these persons require from fifty to five hundred dollars in advance and some of them require the patient to sign a paper which they suppose will relieve them from all obligation to refund, in case the treatment is not satisfactory. We have seen several persons, who looked like sensible, respectable people, who confessed to have "so signed;" we certainly think that they ought to have Catarrh of the nose, head, liver, lights, paunch and gizzard for the remainder of the term of their natural life; we would advise our readers before paying and "signing," to "try it" on a small scale and venture a dollar or two with Prince or Burrington, or rather venture nothing but a little time, on Godfrey who was not afraid to tell us what his Remedy was and shows his faith in its merits by offering to return the money in full on demand "and no questions asked" if it is not satisfactory, and as we know the Remedy and that it is relied upon by educated medical men as safe and efficient in many cases, we rather give our vote for Godfrey first; ☞ if you should find that your family physician does not afford you the desired relief: but to purchase any secret remedy, before consulting a regular physician, merely from the assurances of the persons who sell it, proves that there is something wrong in the upper story, a soft place somewhere in that individual's skull cap.

